# Changes in haematological indices among children with sickle cell disease on hydroxyurea treatment for at least 1 year: A cohort study

**DOI:** 10.1371/journal.pone.0335617

**Published:** 2025-11-03

**Authors:** Stephen Emuli, Crispus Tegu, Faith Oguttu, Ritah Nantale, Paul Ochieng, George Passi, Julian Abeso, Joan Wamulugwa, Milton W. Musaba, Ruth Namazi, Sarah Kiguli, David Mukunya

**Affiliations:** 1 Department of Paediatrics and Child Health, Busitema University, Mbale, Uganda; 2 Department of Community and Public Health, Busitema University, Mbale, Uganda; 3 Department of Anaesthesia and Critical Care, Busitema University, Mbale, Uganda; 4 Department of Paediatrics and Child Health, Mbale regional referral hospital, Mbale, Uganda; 5 Department of Obstetrics and Gynaecology, Busitema University, Mbale, Uganda; 6 Department of Paediatrics and Child Health, Makerere University, Kampala, Uganda; University of Illinois at Chicago, UNITED STATES OF AMERICA

## Abstract

**Background:**

Sickle cell disease is the 12^th^ cause of under-five mortality in Africa, with over 81,000 deaths attributed to sickle cell disease annually. Hydroxyurea is one of the few disease-modifying therapies available for the management of sickle cell disease. This study aimed to assess changes in haematological indices among children who had been initiated on hydroxyurea for at least one year in a non-trial setting at a regional referral hospital in Eastern Uganda.

**Methods:**

We conducted a cohort study, which included children who attended the sickle cell clinic from 21/Aug/2024 to 30/Oct/2024. Data were analyzed using Stata version 18.0. We conducted a paired sample t-test comparing the haematological indices of children with sickle cell disease at baseline and at least one year later.

**Results:**

We included 324 children. Nearly half 155/324 (47.8%) of the participants had good monthly adherence to hydroxyurea. The mean haemoglobin level at follow-up increased by 0.77g/dl (p <0.001) from 7.07g/dl (SD 0.10) at baseline to 7.84g/dl (SD 0.09). There was an increase in the mean corpuscular volume [0.97fl (p=0.645)] and mean corpuscular haemoglobin [0.58pg (p=0.120)]. The white blood cell count decreased by 6.17x10^3^/µl (p<0.001) from 20.77x10^3^/µl (SD ± 0.806) at baseline to 14.60x10^3^/µl (SD ± 0.613) at follow-up. The differential white cell counts of neutrophils, lymphocytes, monocytes, and basophils also decreased.

**Conclusion:**

Hydroxyurea resulted in an increase in mean haemoglobin level and a decrease in absolute and differential white blood cell count. The benefit was more pronounced among children with good adherence to hydroxyurea. We add our voice to calls for continued advocacy for the availability of hydroxyurea for use by children with sickle cell disease in low-resource settings. We also recommend a routine complete blood count to monitor response to treatment and also guide patient management among children with sickle cell disease initiated on hydroxyurea.

## Introduction

Globally, half a million babies are born with Sickle Cell Disease (SCD) each year, and 80% of them are in Africa [1]. Sickle cell disease is the 12^th^ cause of under-five mortality in Africa, with over 81,000 deaths attributed to SCD annually [1]. Children with SCD in Africa are 11 times more likely to die secondary to complications of SCD as compared to their counterparts in high-income countries [2]. Various efforts including newborn screening, penicillin prophylaxis, folic acid supplementation, and vaccination to alleviate morbidity and mortality secondary to SCD, have been implemented but mortality due to SCD is still high [3]. Hydroxyurea (hydroxycarbamide) is one of the few disease-modifying therapies available for the management of sickle cell disease [4].

The primary mechanism of action by which hydroxyurea is effective in individuals with SCD is through the induction of production of fetal haemoglobin, which results in decreased production of sickled haemoglobin. Other suggested mechanisms of action of hydroxyurea include: suppression of bone marrow activity resulting in reduced production of other cells such as white blood cells and platelets, inhibition of ribonucleotide reductase, which results in arrest of maturation of developing cells, and finally, it is metabolised to produce nitric oxide [5]. Production of red blood cells containing fetal haemoglobin reduces the formation of sickle-shaped cells, resulting in improved oxygen carrying capacity of blood, reduced vaso-occlusive episodes, hemolysis, reticulocytosis, and stable erythropoiesis. Nitric oxide produced after metabolism of hydroxyurea acts as a vasodilator, which improves blood flow and reduces adhesion of sickled cells to endothelium [5]. Due to the diverse effects of hydroxyurea as a therapeutic agent, regular monitoring of haematological cell lines is imperative to assess response to treatment, optimal dosing, effectiveness, and any bone marrow suppression.

A number of clinical trials have proven the efficacy and safety of hydroxyurea among children with sickle cell anemia in sub-Saharan Africa [6–9]. Improved haemoglobin levels and mean corpuscular volume, mild bone marrow suppression indicated by reduced white blood cell count, absolute neutrophil count, and reticulocyte count have been reported among children taking hydroxyurea treatment [6,7]. However, hardly any studies have been done among children in the general population outside a trial setting to assess the therapeutic effect of hydroxyurea. Outside a clinical trial setting, adherence and monitoring of lifesaving drugs such as hydroxyurea is not as stringent due to health system factors and social challenges faced by the patients [10]. There is a need to generate data to inform routine monitoring of patients for response to treatment in a normal clinical setting.

The Ministry of health in Uganda added hydroxyurea to the list of essential medicines in 2016 [11]. Hydroxyurea is supplied to national and regional hospitals where children can access the drug through the hospital-based sickle cell clinics. Since the national implementation of the drug, minimal efforts to monitor the effect of the drug outside a clinical trial setting have been fully implemented. There is need to further understand the haematological profiles of children initiated on hydroxyurea so as to guide management, follow up, and optimize treatment protocols in low-resource settings. We conducted a cohort study to assess the changes in haematological indices among children who had been initiated on hydroxyurea for at least one year at a regional referral hospital in Eastern Uganda.

## Methods

### Study design

We conducted a cohort study. We included children who attended the sickle cell clinic during the study period from 21/Aug/2024–30/Oct/2024.

### Study setting

The study was conducted at the Sickle cell clinic of Mbale regional referral hospital. Mbale regional referral hospital is a public hospital in Eastern Uganda, serving a population of about 4.5 million people from over 16 districts in the region, and provides obstetrics and gynaecology, paediatrics, surgery, and internal medicine services. The paediatrics department runs a sickle cell clinic every Wednesday, where over 800 children were registered at the time of the study. All children enrolled in the clinic are of genotype HBSS [8]. Children are routinely reviewed, have their drug refills, and are readmitted if necessary.

### Study population

We included 324 children aged 2–17 years, with a confirmed diagnosis of sickle cell anaemia. We included children with a baseline complete blood count prior to initiation of hydroxyurea. Children not initiated on hydroxyurea or who had been taking hydroxyurea for less than 1 year were excluded.

### Sampling and sample size

Given our recruitment period and target population, we anticipated to recruit 320 participants. With this number, we would have 90% power to detect differences of 0.18 g/dl and above in haemoglobin (our primary outcome), assuming that our baseline haemoglobin was 7.0g/dl and standard deviation was 1 (Stata code: power pairedmeans 7, n(320) power(0.9) sddiff [1]). We consecutively recruited the eligible children attending the sickle cell clinic from 21/Aug/2024–30/Oct/2024 when the required sample size was accrued.

### Study procedures

Data were collected by a trained research assistant using a structured questionnaire. A blood sample was picked and taken to the laboratory for processing. Patient records were reviewed to obtain data for baseline blood count.

### Sample collection and processing

A venous blood sample was taken from the study participant in the procedure room and kept in a purple top vacutainer. The samples collected were taken to the Mbale Regional Referral Hospital main haematology laboratory for analysis within 1 hour. The Sysmex automated Complete Blood Count machine was used. Controls were first run to ensure appropriate calibration of the machine. The sample in the vacutainer was first shaken, then inserted into the sample holder of the machine for analysis, within 3 hours of collection. The printouts of the results were collected by research assistants and entered into the data collection form designed in Kobo Toolbox.

The authors did not have access to information that could identify individual participants during or after data collection.

### Ethics

We obtained ethical approval from the Busitema Research and Ethics Committee, approval number BUFHS REC-2014–180.

We obtained informed written consent from all the caretakers and assent from children aged 12–17years.

### Data analysis

Data from Kobo toolbox were exported to an Microsoft Excel spreadsheet and imported into Stata version 18.0 (StataCorp) for analysis. Descriptive statistics were computed for the dependent and independent variables. We conducted a paired sample t-test comparing the haematological indices of children with sickle cell disease at baseline and the current haematological indices.

Variables: The outcome variables were changes in red blood cell indices (red blood cell count, haemoglobin level, mean corpuscular volume, and mean corpuscular haemoglobin), white blood cell indices (white blood cell count, neutrophils, lymphocytes, monocytes, basophils, and eosinophils), and platelet count. The main exposure variable was initiation on hydroxyurea.

## Results

**Fig 1:**
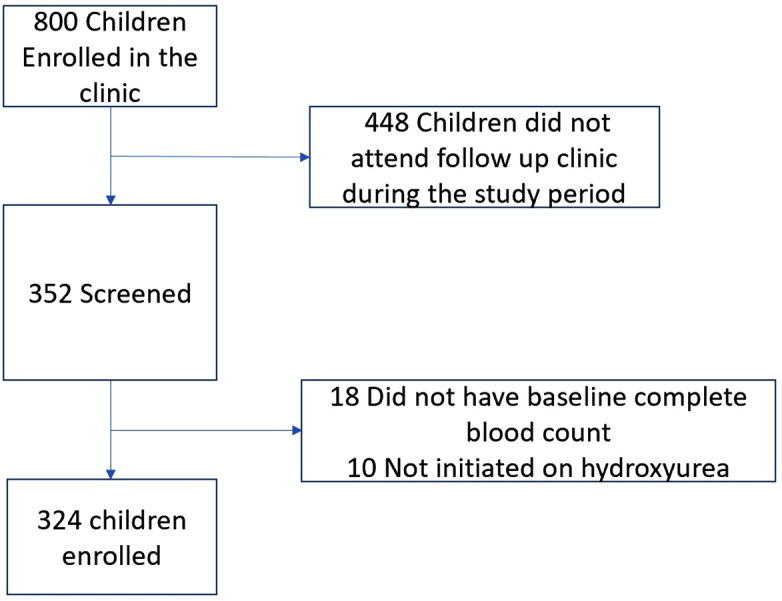
A flow chart showing screening and inclusion criteria for a study among children attending a sickle cell disease clinic.

We screened 352 children and enrolled 324 children. Reasons for exclusion are detailed in Fig 1.

Nearly half 47.8% (155/324) of the participants were male, almost half of the children 48.1% (156/324) were aged 6–12 years, and the median age was 10.5 years (IQR 7–13). Details are in Table 1.

**Table 1:** Sociodemographic characteristics of children with sickle cell disease initiated on hydroxyurea for at least one year.

	Total (frequency) N = 324
Age of child	
≤ 5 years	63 (19.4)
6–12 years	156 (48.1)
13–17 years	105 (32.4)
Gender	
Male	155 (47.8)
Female	169 (52.2)
Place of residence	
Rural	224 (69.1)
Urban	100 (30.9)
Distance to facility in Km	
≤ 5	90 (27.8)
> 5	234 (72.2)
Caretaker of child	
Both parents	248 (76.5)
Single parent	42 (13.0)
Other relatives	34 (10.5)
Education level of the caregiver	
No formal education	11 (3.4)
Primary	161 (49.7)
Secondary	93 (28.7)
Tertiary	59 (18.2)

Nearly half 155/324 (47.8%) of the participants had good monthly adherence to hydroxyurea, 85/324 (25.3%) missed hydroxyurea for less than 7 days in a month, 29/324(8.95%) missed hydroxy urea for more than 7 days in a month and 58/324 (17.9%) missed hydroxyurea for more than a month, 244/324 (75.3%) had malaria in the past one year and 142/324 (43.8%) had received a blood transfusion over the past one year. Details are in Table 2.

**Table 2:** Clinical characteristics of children with sickle cell disease initiated on hydroxyurea for at least one year.

	Total (frequency) N = 324
Received blood transfusions in the last 1 year	
Yes	142 (43.8)
No	182 (56.2)
Hospitalized in last 12 months	
Yes	242 (74.7)
No	82 (25.3)
Occurrence of painful crises in last year	
Yes	283 (87.3)
No	41 (12.7)
Malaria in the last one year	
Yes	244 (75.3)
No	80 (24.7)
Hydroxyurea adherence monthly	
Very good (did not miss a day)	155 (47.8)
Good (missed < 7 days in a month)	82 (25.3)
Poor (missed ≥ 7 days in a month)	29 (8.95)
Very poor (missed > 30 days)	58(17.9)

### Changes in haematological indices among children with sickle cell disease, after initiation of hydroxyurea for at least one year

#### Red blood cell indices.

The mean haemoglobin level at follow-up increased by 0.77g/dl (p < 0.001) from 7.07g/dl (SD 0.10) at baseline to 7.84g/dl (SD 0.09). The mean corpuscular volume (MCV) increased by 0.977fl (p = 0.645) from 84.42fl (SD 2.09) at baseline to 85.39fl (SD 0.66) at follow-up. The mean corpuscular haemoglobin increased by 0.58pg (p = 0.120) from 29.36pg (SD = 0.36) at baseline to 29.94pg (SD ± 0.22) at follow-up. This is summarized in Table 3.

**Table 3:** Changes in haematological indices among children with sickle cell disease, after initiation of hydroxyurea for at least one year.

	Mean	SD	Mean difference	t	*p*-Value
Red blood cell count (x10^6^/µl)					
Baseline	2.58	0.05	0.13	2.139	0.033
Current	2.71	0.05			
Haemoglobin Level (g/dl)					
Baseline	7.07	0.10	0.77	6.919	<0.001
Current	7.84	0.09			
Mean corpuscular volume (fl)					
Baseline	84.42	2.09	0.97	0.460	0.645
Current	85.39	0.66			
Mean corpuscular haemoconcentration (pg)					
Baseline	29.36	0.36	0.58	1.561	0.120
Current	29.94	0.22			
White blood cell count (x10^3^/µl)					
Baseline	20.77	0.806	−6.17	−7.235	<0.001
Current	14.60	0.613			
Neutrophils (x10^3^/µl)					
Baseline	9.77	0.55	−4.09	−6.641	<0.001
Current	5.68	0.29			
Lymphocytes (x10^3^/µl)					
Baseline	9.04	0.48	−0.16	−0.067	0.946
Current	8.89	2.27			
Monocytes (x10^3^/µl)					
Baseline	1.82	0.10	−0.57	−5.868	<0.001
Current	1.25	0.04			
Basophils (x10^3^/µl)					
Baseline	0.40	0.08	−0.22	−2.739	0.007
Current	0.18	0.01			
Eosinophils (x10^3^/µl)					
Baseline	0.65	0.10	0.24	1.289	0.198
Current	0.89	0.16			
Platelets (x10^3^/µl)					
Baseline	363.85	9.57	38.19	3.237	0.001
Current	402.04	9.85			

#### White blood cell indices.

The white blood cell count decreased by 6.17x10^3^/µl (p < 0.001) from 20.77 x10^3^/µl (SD ± 0.806) at baseline to 14.60 x10^3^/µl (SD ± 0.613) at follow-up. The neutrophil count decreased by 4.09 x10^3^/µl (p < 0.001) from 9.77 x10^3^/µl (SD ± 0.55) at baseline to 5.68 x10^3^/µl (SD ± 0.29) at follow-up. Lymphocytes, monocytes and basophils also decreased. Details are summarized in Table 3.

#### Platelets.

The platelet count increased by 38.19 x10^3^/µl (p = 0.001) from 363.85 x10^3^/µl (SD ± 9.57) at baseline, to 402.04 x10^3^/µl (SD ± 9.85) at follow-up.

### Changes in haematological indices among children with sickle cell disease, after initiation of hydroxyurea for at least one year, stratified by adherence

Overall, the children who had good adherence to hydroxyurea (missed less than 7 days a month) had a more marked increase in the red blood cell indices and a more marked decrease in the white blood cell indices as compared to children with poor adherence to hydroxyurea. Details are in Table 4.

**Table 4:** Changes in haematological indices among children with sickle cell disease, after initiation of hydroxyurea for at least one year, stratified by adherence.

	Good adherence	Poor adherence	Good adherence	Poor adherence
	Mean count		Mean difference (SD)	
Red blood cell count (x10^6^/µl)				
Baseline	2.56	2.62	0.15(1.19)	0.07(0.93)
Current	2.71	2.69		
Haemoglobin Level (g/dl)				
Baseline	7.05	7.11	0.82(1.93)	0.61(2.14)
Current	7.87	7.73		
Mean corpuscular volume (fl)				
Baseline	82.72	81.7	4.09(17.20)	0.22(13.55)
Current	86.82	81.47		
Mean corpuscular haemoconcentration (pg)				
Baseline	29.5	28.96	0.76(6.48)	0.07(7.28)
Current	30.27	29.04		
White blood cell count (x10^3^/µl)				
Baseline	20.66	21.03	−6.21(16.36)	−6.02(12.22)
Current	14.44	15.01		
Neutrophils (x10^3^/µl)				
Baseline	9.95	9.25	−4.44(12.05)	−3.12(7.78)
Current	5.51	6.13		
Lymphocytes (x10^3^/µl)				
Baseline	8.61	10.22	−1.97(11.18)	−2.75(11.62)
Current	6.64	7.46		
Monocytes (x10^3^/µl)				
Baseline	1.72	2.06	0.53(1.67)	−0.67(1.94)
Current	1.196	1.38		
Basophils (x10^3^/µl)				
Baseline	0.33	0.57	−0.14(0.90)	−0.41(2.37)
Current	0.18	0.16		
Eosinophils (x10^3^/µl)				
Baseline	0.7	0.49	0.25(3.88)	0.21(1.24)
Current	0.95	0.7		
Platelets (x10^3^/µl)				
Baseline	365.67	358.88	33.36(227.12)	51.35(166.20)
Current	399.03	410.24		

## Discussion

We noted an increase in the red blood cell indices: red blood cell count, haemoglobin level, mean corpuscular volume, mean haemoconcentration, and platelet count. There was a decrease in the white blood cell indices: white blood cell count and differential white cell count (neutrophils, lymphocytes, monocytes, basophils, and eosinophils). All these changes were more pronounced among children with good adherence to hydroxyurea.

### Increase in red blood cell indices

Increase in red blood cell count is due to the fact that hydroxyurea induces improved erythropoiesis with the production of more stable red blood cells rich in fetal haemoglobin [12]. These red blood cells are less liable to haemolysis, grow to maturity, and this allows a higher red blood cell count to accumulate in circulation [5]. The reduction in haemolysis and increased life span of the fetal haemoglobin-rich red blood cells also results in the observed increase in the level of haemoglobin. The newly formed red blood cells have a larger diameter, which results in an increase in mean corpuscular volume (MCV). Hydroxyurea also causes mild bone suppression, this reduces the production of smaller reticulocytes and leads to a relative increase in larger mature red blood cells, hence increased MCV.

The average amount of haemoglobin per red blood cell (Mean Corpuscular Haemoglobin (MCH)) increases due to the fact that the newly produced red blood cells have an increased lifespan, which allows them to mature and results in more stable red blood cells with high haemoglobin concentration. Other studies in Uganda have also reported an increase in red blood cell indices such as; – red blood cell count, level of haemoglobin, MCV, and MCH among children after initiation of hydroxyurea [6–8]. Studies in other countries such as India [13,14], Iran [15], and America [16] have also reported similar findings. This similarity in findings can be explained by the fact that the mechanism of action of hydroxyurea is the same in all patient populations.

### Decrease in white blood cell indices

Decrease in white blood cell count is due to the cytoreductive effect of hydroxyurea [17]. Hydroxyurea reduces proliferation in the bone marrow by inhibiting the ribonucleotide reductase required for Deoxyribonucleic acid (DNA) synthesis. This results in decreased production of myeloblasts, hence a decrease in white blood cell count and decreased differential white cell count [18]. Children started on hydroxyurea have reduced sickling and haemolysis as well as improved endothelial function [19]. This results in reduced systemic inflammation and a reduced immune stimulation for the production of white blood cells [16]. Our findings are supported by other studies that have also reported a decrease in absolute and differential white blood cell count among children with SCD taking hydroxyurea [14,16,20]. This similarity in findings can be explained by the fact that the mechanism of action of hydroxyurea is the same in all patient populations.

These observed changes in red blood cell and white blood cell indices can be used as indicators of hydroxyurea efficacy in children with SCD in a routine clinical setting. Clinicians can use these indices to assess response to therapy and adjust dosing appropriately.

### Increase in platelet count

Our study also noted a significant increase in mean platelet count after initiation of hydroxyurea. This was contrary to the expected myelosuppressive effect of hydroxyurea on platelet production. This is also contrary to other studies, which have reported a decrease in platelet count among patients with hydroxyurea [14] and reports of thrombocytopenia as a known side effect of hydroxyurea [16]. This paradoxical increase of platelets among patients taking hydroxyurea has also been reported among children with SCD in the United States [21], Saudi Arabia [22], and Brazil [23]. The noted increase in platelet count may be due to individual variations in bone marrow response to hydroxyurea [24]. Among patients still in the early phase of hydroxyurea therapy initiation or receiving suboptimal doses of hydroxyurea, the drug may not cause enough myelosuppression to reduce platelet production.

### Haematological response stratified by adherence to hydroxyurea

Therapeutic benefits such as an increase in red blood cell indices and a decrease in white blood cells were more marked among children with good adherence to hydroxyurea. The changes in red blood cell indices and white blood cell indices were nonetheless suboptimal in comparison to changes observed among children enrolled in the NOHARM-MTD trial [25]. This can be explained by the fact that in a trial setting, participants have better adherence due to an adequate supply of hydroxyurea. Drugs are prescribed and dispensed more accurately in a trial setting, and dose escalation is also done with adequate monitoring.

In our study, good adherence was assessed by word of mouth which is unreliable, and good adherence was considered if a participant had missed less than 7 days in a month. Hydroxyurea is prescribed at 15–25 mg/kg and dispensed to the closest 500 mg capsule for age, therefore children receive suboptimal doses. This is minimized in a clinical trial setting where tablet formulations are dispensed more accurately and dosing can be increased to 30 mg/kg.

These findings highlight the need to emphasize adherence to hydroxyurea even in routine clinical settings. We also recommend and advocate for closer monitoring and titration of hydroxyurea to maximum tolerated doses in routine clinic settings.

### Strengths and limitations

We ably collected baseline data and follow-up data a year later for comparison. This is one of the first studies to be done in our routine clinical setting versus a clinical trial. Data for adherence to hydroxyurea was elicited from history obtained from caregivers, this is unreliable and subject to bias due to social desirability. We did not collect medical history about blood transfusions and admissions one year prior to initiation of hydroxyurea, therefore, we could not compare these characteristics to assess the efficacy of hydroxyurea. We also did not collect data about nutrition and supplements such as folate, which affect haemoglobin levels among children. This was a single-site study, so our findings may not be generalizable to the general population.

## Conclusion

Our study found that hydroxyurea was of therapeutic benefit to the children, as evidenced by an increase in mean haemoglobin level and a decrease in white blood cell count. The benefit was more pronounced among children with good adherence. We recommend continued advocacy for the availability of hydroxyurea for use by children with sickle cell disease in low-resource settings. We also recommend a routine complete blood count to monitor response to treatment and also guide patient management among children with sickle cell disease initiated on hydroxyurea.

## Supporting information

S1 Data (XLS)
